# Utilisation of after-hours telephone support in a home-based hospice service

**DOI:** 10.1186/s12904-022-01049-5

**Published:** 2022-09-16

**Authors:** Poh-Heng Chong, Jasmin Lee, Zhi-Zheng Yeo, Raymond Qishun Ang

**Affiliations:** HCA Hospice Limited, 705 Serangoon Road, #03-01, Block A @ Kwong Wai Shiu Hospital, 328127 Singapore, Singapore

**Keywords:** After-hours, Phone call, Home-based, Logistic regression, Emergency outcomes

## Abstract

**Background:**

After-hours support from hospice providers is instrumental to patients with serious illness who choose to remain at home, particularly at end of life. Utilisation of out-of-hours support has been much characterised in terms of frequency and nature of calls, but more needs to be known to inform service customisation and resource allocation to optimise care. To this end, we stratify reasons for using the after-hours helpline according to time sensitivity, and to explore disease and person factors associated with urgent calls.

**Method:**

Electronic medical records for incoming calls from external parties outside workhours within a large home hospice in Singapore were analysed inductively, to identify patterns and associations along study objectives. Individual code books for caller type and call reasons were created and tested in vivo, and later administered to extracted data. Patients that accessed the helpline were tracked for different outcomes, including hospital admissions and on-call home visits. Logistic regression modelling was performed to categorise call reasons by urgency and to identify disease and person factors associated with time sensitive calls.

**Results:**

More than 5,000 calls to the helpline were made over a two-year period (2019-2020), predominantly by family caregivers (88.4%). These were in relation to 2,303 unique patients (38.9% of total patients served). After-hours calls were made an average of 2.3 times by patients across various lengths of service. Only 11.9% of calls were deemed time sensitive or urgent, requiring home visits by on-call staff (4%) or resulting in admission to hospital (7.9%). The majority were managed by primary care teams on the next workday (65.1%) and the remainder sorted during the after-hours call itself (22.3%). Call reasons or presenting issues were classified into two groups according to urgency. Calls in the year 2020, from the younger patient, preferred place of death outside the home, and caller types other than patient or healthcare worker were significantly associated with urgent calls.

**Conclusion:**

Deeper characterisation of after-hours calls offers possibilities: service redesign for optimal resourcing and customised training for better care. Ultimately, planners, providers, and patients all stand to benefit.

**Supplementary Information:**

The online version contains supplementary material available at 10.1186/s12904-022-01049-5.

## Background

After-hours palliative care support is a lifeline to patients with serious medical conditions who choose to spend more days at home rather than an institution like hospital or hospice [1–8]. Operating a 24/7 telephone helpline is fundamentally how most services render out of hours support. Resource considerations manning these helplines and its toll on designated healthcare staff have been acknowledged [9, 10], but current evidence reveals little beyond different reasons for seeking urgent assistance while receiving hospice support at home [7, 11–16]. It is unclear how individual calls outside normal work hours are managed in a full suite service that also provides emergency home visits. Patients’ disposition and outcomes afterwards are also rarely tracked. Mapping process-related data longitudinally can inform strategic service development to optimise utilisation or reduce misuse, that ultimately enhances holistic care of these patients and caregivers [2, 5, 13, 17, 18].

Access to an after-hours helpline may not seem a core service element as patients and families are first introduced to hospice home care, but it does become critical to users over time. The period in question — after-hours — constitutes up to three quarters of the week or even year [2, 3, 5]. Yet, it is often manned by a tiny fraction of the supporting clinical team in settings where this service stream is under the purview of palliative care. In the context of end-of-life care, almost 30-40% of families have used this service [5, 17]. It is also a major determinant for location of death in this patient group [1]. In relation to after-hours support, the literature has flagged identifying relevant stakeholder characteristics and best ways to help dying patients remain in their place of choice as research priorities. (Shabnam et al 2018 & Smith et al 2015) At the family-unit level, particularly with uncontrolled symptoms or sudden changes in condition of the seriously-ill patient, after-hours support has proven pivotal in allaying fear and anxiety among caregivers at home [4].

Healthcare providers however are divided on whether after-hours support rendered by an on-call team is utilised appropriately. While a few services have reported underuse [2, 4, 16], most argue usage could be better directed [1, 2, 11, 12, 19]. A service audit within a large hospice agency in the United States revealed only 30% of after-hours calls were clinically related, and almost 40% were deemed avoidable [12]. The latter included requests for supplies and medication refills. Ineffective service implementation not only raises healthcare expenditure but dampens staff satisfaction [9, 13]. In fact, after-hour duties have been cited as a major reason why healthcare workers shun away from hospice and palliative care work [10]. Community hospices in Singapore have faced similar challenges; mitigating measures like capping phone support and unscheduled home visits after midnight have been applied when staffing became problematic.

Given instances of suboptimal utilisation outlined and considerations of cost effectiveness and service sustainability, rather than focusing on reasons for calls to the helpline [12–14, 16], exploring dispositions or outcomes thereafter could reveal hidden opportunities for service improvement. Diverse reasons for accessing a helpline may be stratified by urgency, like whether an emergency home visit or hospital admission occurred. It addresses questions around what may be ‘valid’ reasons for calling and which groups of users are associated with them. Findings offer the potential for hospice administrators or programme managers to customise or redesign after-hours support at home as a complex healthcare intervention within community palliative care [20, 21].

### Context

This study is conducted in the largest home hospice service in Singapore. The home care service is primarily nurse-led and supports more than half of the country’s home-based palliative and hospice care needs. It is backed by a full suite of multi-disciplinary professionals like physicians, nurses, social workers and various therapists. All service elements, including after-hours support, are complimentary to patients with life limiting illness, till discharge or death.

For the purpose of this study, after-hours refers to the period from 6.00pm to 8.30am the next morning on weekdays and round the clock on Saturdays, Sundays and eleven gazetted Public Holidays in Singapore. A dedicated helpline shared with all registered patients (around 800 patients anytime) is manned by on-call hospice clinical staff based on a rota. One hospice nurse performs on-call duty from 830am to 6pm during weekends and public holidays, while one doctor takes evening calls on all weekdays, weekends, and public holidays (from 6pm to 830am next day). While patients and families are instructed to use the helpline for prompt assistance outside office hours, it is emphasized that this facility should not replace the national hotline for medical emergencies (995).

Support through phone advice is primarily intended, though home visits are sometimes made for on-site clinical assessment and management at home when clinically indicated. Examples of these situations include uncontrolled symptoms despite administration of breakthrough medications or unexpected deterioration of a patient who had wanted no more hospital admissions. All documentation related to after-hours calls (and on-call visits) are recorded in the organisation’s electronic medical records. Clinical notes will be read by the patient’s primary hospice nurse on the next working day, upon an email alert.

## Methods

We studied data in electronic medical records from staff performing on-call duties over a two-year period (2019 – 2020). An inductive approach was adopted to analyse documentation associated with incoming calls to the helpline for common patterns. We hypothesised that calls with emergency outcomes like hospital admissions or unplanned after-hours home visits are urgent, and these may be associated with patient characteristics (e.g., demographics), their preference for place of death, and the type of caller.

### Inclusion and exclusion criteria

We accessed case notes for all incoming phone calls after-hours made by external parties to the hospice helpline between 1^st^ January 2019 and 31^st^ December 2020. Outgoing calls made by hospice personnel to patients or other persons were excluded, as these calls were primarily related to follow-up of routine work performed during the day, and did not constitute emergencies or issues occurring after office-hours.

### Procedures

Demographic and other relevant information for unique patients (some had called the helpline more than once) were extracted: age, sex, ethnicity, marital status, primary diagnosis, preferred place of death, and length of service. This group of patients who made after-hours calls was compared against the other group that never made after-hours calls to determine if there were differences in characteristics.

As there were no structured fields to input data related to after-hours calls before, documentation from electronic medical records was all captured in free form text. Other than broad categorisations like ‘clinical’ or ‘non-clinical’, the existing literature did not provide useful guidance to organise the dataset. Hence, before final data analysis, empirical manual coding of abstracted case notes was performed.

For this task, the research team created two codebooks. The first codebook was for ‘types of callers’ to describe their relation to each patient. We created eight groups to categorise all possible callers, as shown in Table 1.

**Table 1:** Codebook for categorizing callers

Child		The child of the patient, related by blood or by law (e.g., Son-in-Law; Daughter-in-Law).
Partner		Includes either the spouse of the patient, or the patient’s long-term partner.
Parent		Parent of the patient, by blood or by law.
Extended family		Includes other family relations to the patient, such as niece/nephew, grandchildren, or siblings.
Healthcare worker		Patient’s healthcare provider from other institutions, e.g., specialist, private nurse, nursing aide.
Patient		Patient him/her-self is the caller.
Live-in helper		Dedicated domestic helper employed to care for the patient.
Others		Includes patients’ acquaintances e.g., friends, neighbours, landlords/tenants.

The second codebook was conceived to capture myriad reasons for after-hours calls. An initial list (with definitions and keywords) was first generated by Resident Physician JL and Senior Consultant in Palliative Medicine PHC after early thematic coding of six month’s extracted data. This list was reduced and refined over subsequent research meetings, informed in parts by extant literature, practice experience and study objectives (see Supplemental file 1).

All study documentation extracted from electronic medical records within the study period was eventually coded by Resident Physician JL, Research Executive ZZY, and Research Assistant RA, using Excel 2016. Before commencement, the group calibrated their coding to improve inter-rater reliability. A subset of 100 randomly selected case notes were selected; members independently coded each note, and then compared the results. For each case note where there were disagreements (i.e., no unanimous code), the group discussed and then adjusted their coding. This was repeated until there was unanimous agreement (i.e., percent agreement = 100%). Subsequently, each member coded equal portions of the remaining dataset.

Third, we reviewed follow-up actions after each call to the helpline. To track follow-up and other outcomes, we analysed service utilisation data after calls were received; they included home visits made by duty personnel or the patient’s primary nurse, phone calls, hospital admissions, and occurrence of death. There were five possible outcomes (with increasing intensity of support):

(i) Non-Emergency outcomes

- No follow-up (issue was resolved during the call),

b. Primary nurse made a follow-up phone call on the next working day,

iii. Primary nurse conducted a home visit on the next working day,

(ii) Emergency outcomes
(iii) On-call staff made an emergency home visit to the patient,

e. Patient was admitted to hospital.

We assumed that ‘reasons for calls’ may be stratified into calls with urgent versus non-urgent issues by examining their association with the outcomes. Logistic regression was employed to model probability of emergency/non-emergency outcomes against “reasons for calls”; The code “clarification and confirmation” was used as the reference category – reasons associated with higher likelihood for emergency outcomes than the reference category would be classified as urgent and the rest non-urgent.

Subsequently, using the stratified calls as outcome, forward-conditional logistic regression was employed to identify factors that predicted likelihood of the call’s urgency. Variables were iteratively added to the model and only kept if it provided statistically significant improvement, otherwise they were dropped from the final model. Variables tested included patient demographics, relationship of caller to patient, year of the call (2019 or 2020), and preferred place of death. These covariates were informed by extant literature (including gaps raised) and arrival of the COVID pandemic in early 2020. To better inform service-planning through key predictors, we had sought to produce a less complex model with fewer covariates. Hence, an inclusion cut-off of α = 0.05 was used.

Relevant cross-tabulations and statistical tests (i.e., Χ^2^ test, t-test, binary logistic regressions) were conducted using IBM SPSS 25.

### Research ethics

This study was a non-interventional, retrospective study using data that was collected as part of service provision. No information was directly collected from human participants for the conduct of this study. This study was reviewed by the SingHealth Centralised Institutional Review Board (Singapore) on 3rd May 2021; the board determined that research ethics approval was not required.

## Results

**Fig. 1:**
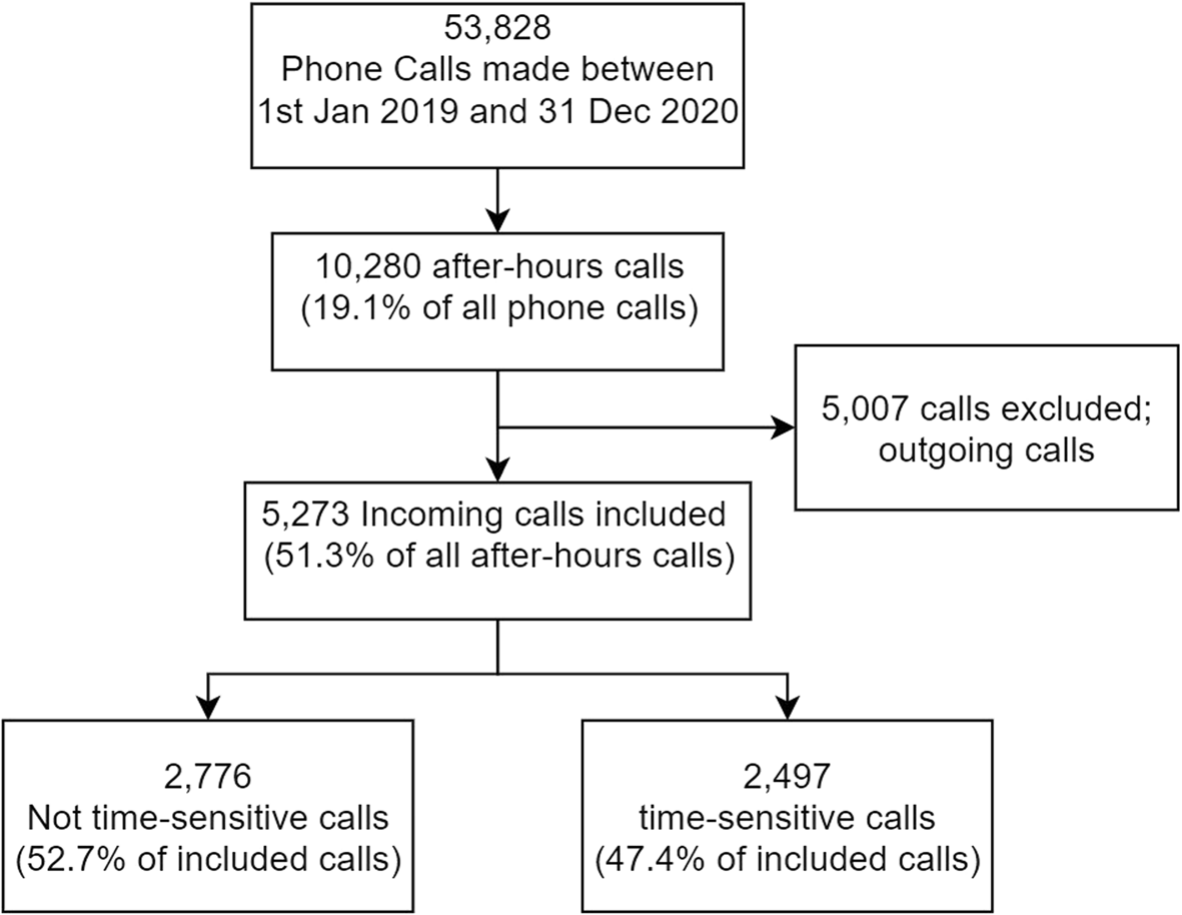
Breakdown of inclusion process for phone call case notes

Figure 1 shows a breakdown of all incoming phone calls, and calls after hours on the helpline. 53,828 calls were received over the study period of two years. Of this number, 10,280 (19.1% of all calls) were annotated as “After Office Hours” calls. After reviewing notes to identify only incoming calls from external parties, 5,273 after-hours calls were finally included for in-depth analysis. 2,391 after-hours calls were received in 2019, and 2,882 in 2020.

### Patient Characteristics

After-hours calls were made in relation to 2,303 individual patients (38.9% of 5,951 total patients served between 2019 and 2020). As mentioned, this group was compared against remaining patients who did not call. Significant differences were found between both groups for patients’ age, ethnicity, and marital status. First, patients associated with after-hours calls appeared to be older. Second, compared to the group that never made any after-hours calls, there was a higher proportion of Chinese patients and a lower proportion of Malay patients. Finally, there were less patients who were single in the after-hours calls group (Table 2).

**Table 2:** Comparison of patient characteristics – who use the after-hours service and those who don’t

Total no. of patient served (2019-2020) (*N*=5939)	Made after-hours calls (*n*=2,303)	Did not make after-hours calls (*n*=3,636)	*p*-value
Gender			
Male (%)	1120 (48.6%)	1795 (49.4%)	N.S.
Female (%)	1183 (51.4%)	1841 (50.6%)	
Mean age in years (SD)	75.6 (12.4)	74.0 (12.7)	.000*
Length of service-days			
Range	1 - 2839	1-2213	N.S.
Median	65	58	
IQR	27 - 160	19-164	
Diagnosis group			
Cancer (%)	1863 (80.9%)	2886 (79.4%)	N.S.
Non-Cancer (%)	440 (19.1%)	750 (20.6%)	
Ethnicity			
Chinese (%)	1850 (80.3%)	2833 (77.9%)	.031*
Malay (%)	218 (9.5%)	445 (12.2%)	.001*
Indian (%)	119 (5.2%)	173 (4.8%)	*N.S.*
Others (%)	116 (5.0%)	185 (5.1%)	*N.S.*
Marital Status			
Married (%)	1427 (62.0%)	2224 (61.1%)	N.S.
Widowed (%)	620 (26.9%)	922 (25.4%)	N.S.
Single (%)	149 (6.5%)	298 (8.2%)	.014*
Divorced/Separated (%)	107 (4.6%)	192 (5.3%)	N.S.

*p < 0.05 at 95% confidence

N.S, not significant, *p* > 0.05

**Fig. 2:**
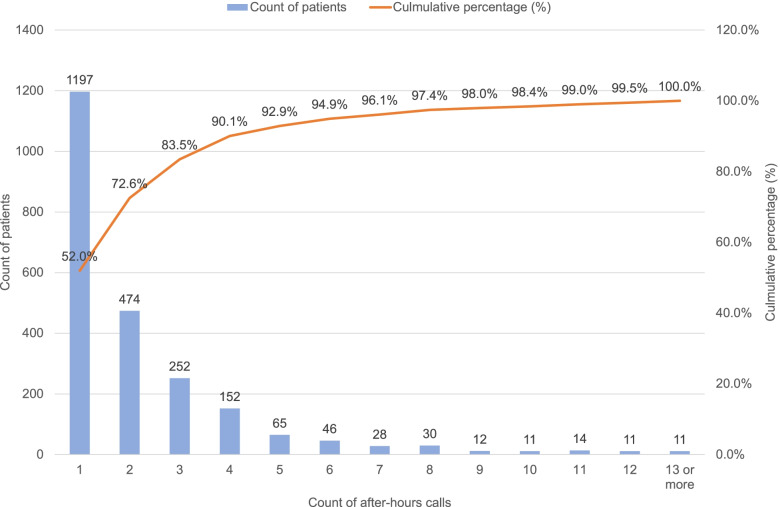
Count of patients by number of after-hours calls made (*n*=2,303)

Figure 2 shows patient distribution by number of after-hours calls made. The median number of after-hours calls made was 1 (IQR: 1-3); more than 80% who used the helpline made three or less calls.

### Call characteristics

Of 5,273 incoming calls after hours over two years, 88.4% were made by family caregivers (inclusive of children, partners, extended family and parents). The remaining calls were from healthcare workers, the patient him/her-self, live-in helpers, or other individuals.

### Modelling follow-up outcomes against reasons for calls

Table 3 shows distribution of cases according to follow-up outcomes. Almost two thirds or 65.1% of patients who called the helpline received follow-up by their own primary nurses either on the phone or through home visits on the next workday. 210 patients (4.0%) required an urgent home visit by on-call personnel, and 414 patients (almost 8%) were admitted to hospital after calls to the hospice helpline.

**Table 3:** Follow-up outcomes for after-hours calls, in order of increasing intensity of support rendered

Follow-up outcomes	Number of calls
No follow-up documented	1178 (22.3%)
Follow-up phone call by primary nurse by next working day	1297 (24.6%)
Visit by the primary nurse by next working day	2174 (40.5%)
Emergency visit by on-call team	210 (4.0%)
Hospitalized on the same day	414 (7.9%)

We conducted logistic regression for follow-up outcomes (emergency or non-emergency) against reasons for calls. Reasons that were associated with higher likelihood of emergency outcomes (on-call visits or hospital admissions) were pain, breathing-related issues, bleeding, altered mental status, acute deterioration, medical emergencies, and falls or accidents (all *p* <.05). Table 4 presents all coded reasons, grouped according to urgency.

**Table 4:** Reasons for calls, grouped by classification of urgency as identified by logistics regression model

Reasons for the call (*n*=5273)	Number of calls	Odds Ratio	95% Confidence interval	*p*-value
Calls with urgent issues				
Falls or accidents	40	2.59	1.16-5.82	.021*
Bleeding-related issues	152	2.34	1.46-3.75	.000*
Altered Mental State	154	2.41	1.51-3.83	.000*
Medical Emergencies	193	4.91	3.36-7.18	.000*
Fever	307	1.51	1.01-2.27	.047*
Breathing-related issues	418	2.31	1.64-3.23	.000*
Pain	563	1.45	1.03-2.04	.033*
Deterioration or decline	638	1.97	1.44-2.70	.000*
Calls with non-urgent issues				
Service availability & alternative resources	30	0.36	0.05-2.66	.315
Sleep-related issues	31	1.54	0.52-4.50	.433
Adverse reaction to treatment	39	0.56	0.13-2.37	.432
Diarrhoea	74	1.44	0.69-2.99	.333
Medication refill	92	0.60	0.24-1.51	.276
Requesting or Donating equipment	102	0.42	0.15-1.18	.101
Caller distress	106	1.58	0.86-2.90	.141
Skin-related issues	145	0.45	0.19-1.05	.064
Constipation	150	0.98	0.53-1.82	.960
Others	157	1.18	0.67-2.07	.572
Administrative matters only	201	1.08	0.64-1.83	.766
Tube-related issues	208	1.04	0.62-1.76	.875
Informing that patient has died	563	0.30	0.18-0.52	.000*
*Clarification or confirmation* *(Reference Category)*	910	-	-	-

* *p* < 0.05 at 95% confidence

### Modelling urgent calls against patient factors

Table 5 summarises results from the forward-selection stepwise logistic regression model. The final model revealed patients’ age, year of call, preferred place of death, and caller type as significant factors. Variables dropped during statistical analysis included patients’ gender, ethnicity, marital status, and diagnosis group. The following observations were made: (i) Younger patients were more likely to call for urgent issues, (ii) Calls made in 2020 were more likely to involve urgent issues than in 2019, (iii) Patients with preferred place of death other than home were more likely to make calls with urgent issues, and (iv) Calls from other healthcare workers or the patients themselves were less likely to be ‘urgent’ compared to callers who were patients’ children (son or daughter).

**Table 5:** Binary logistic regression for urgency of calls (Forward Conditional)

	Number of calls	Odds Ratio	95% Confidence Interval	*p*-value
Patient’s Age	-	0.99	0.99-1.00	.002*
Year of call				
*2019 (Reference category)*	*2391*	*-*	*-*	*-*
2020	2882	1.22	1.09-1.36	.001*
Preferred Place of Death				.019*
*Own Home (Reference category)*	*2627*	*-*	*-*	*-*
Not known	1714	1.11	0.98-1.26	.096
No preference	605	1.12	0.94-1.34	.214
Others (Hospital/Nursing Home/Hospice)	327	1.44	1.13-1.83	.003*
Caller				.000*
*Child (Reference category)*	*3510*	*-*	*-*	*-*
Partner	582	0.91	0.76-1.10	.340
Extended	548	0.94	0.79-1.13	.534
Healthcare worker	326	0.45	0.35-.59	.000*
Patient	181	0.69	0.50-.94	.019*
Live-in Helper	72	1.49	0.93-2.39	.100
Others	34	0.97	0.49-1.92	.925
Parent	20	1.92	0.72-5.09	.192

* p < 0.05 at 95% confidence

The following variables were dropped from the stepwise regression model: Patient’s gender, Patient’s ethnicity, Patient’s marital status, and Patient’s diagnosis group (Cancer or Non-Cancer)

## Discussion

Important insights to envision rendering of efficacious after-hours palliative care support at home have been obtained. Among more than 50,000 incoming calls to the hospice at home service over a two-year period, one in five were received outside work hours, made approximately by 40% of all patients served. Marginal differences between users and non-users in terms of age, ethnicity and marital status were noted, though their implications are unclear. Callers were predominantly family caregivers, and each family mostly used the helpline 1-3 times (average of 2.3 over length of service). Calls related to emergency home visits or hospitalisations made up 12% or around one in eight of all after-hours calls to the helpline. Specific presenting issues were found to be associated with these emergency outcomes. These observations have different service implications.

Given that the period outside hospice work hours constitute a significant portion of any patient’s care at home, availability of a robust support framework and its appropriate use (or misuse) is understandably a priority to all types of stakeholders, albeit for different reasons. This is particularly relevant for patients receiving palliative care at home, especially near end of life, or when further hospital admissions are against declared goals of care. It is not surprising that many care recipients made use of the helpline to seek assistance of different kinds. We found similar patterns of utilisation in other institutions [5, 17]. Attempted profiling of the patient (and caller) most likely to call for an emergency issue that we performed here could facilitate initial patient triage and subsequent management by the on-call team [13]. The team however still needs to decide whether to make an urgent home visit or summon an ambulance instead. Separately, although only one in eight calls were assessed to be related to emergency issues, the rest were not necessarily unimportant, just less time sensitive. This is where less resource intensive yet targeted solutions may be deployed.

Almost a quarter (22.3%) of callers had their issues sorted promptly on the phone alone, while almost two thirds (65.1%) could wait till the next workday for primary teams to pick up issues raised (through home visits or calls during normal work hours). Staff empowerment (see next section) and training in ‘tele-support’[13] are worthy considerations to consolidate or enhance. In our own experience, even though a helpline is made available after hours, not all families utilise it. But when they do, caregiver fears and anxiety have been assuaged just having someone pick up the call. Besides, timely attention to what is initially perceived as ‘non-emergency’ could avert a real emergency later. Findings substantiate the value to beneficiaries, in services where visits at home after-hours are not part of the overall care package [13]. From the service requirement perspective, we noted that urgent home visits by staff on call were needed only 4% of the time. This reflects projected demand in terms of visits at home anticipated outside office hours, useful information to service leads looking at costing and long-term sustainability.

Focusing on the top reasons for accessing help from providers after hours, we raise two practice points for consideration. First, training of new staff on rota to man the helpline. Eight emergency issues were flagged as prevalent, and several minor medical issues were consistently raised. Given the need to triage for hospital admission (or render initial management) mentioned before and big role of phone-based support (at least till the next workday), customised staff training including resource aides could be produced [7, 12, 17]. Second, like others before [5, 12], work improvement efforts around better ‘in-hours’ communication to reduce the need to ‘clarify care plans’ (17.6% of calls) and deliberate advice on procedures after death to minimise calls ‘informing that patient has died’ (10.5% of calls) are worth looking into [7, 15, 19]. Avoidable calls thus could be managed better, minimising staff frustration in the process.

Finally, the study period provided a unique opportunity to examine the impact of the COVID-19 pandemic on community palliative care. Findings showed that not only were there more after-hours calls made in 2020 (start of the pandemic) compared to 2019, they were also more likely to involve urgent issues. To our knowledge, this study is the first to report the influence of COVID-19 on after-hours calls within the home hospice setting. It highlights yet another way the long-drawn pandemic has impacted health care.

### Study limitations

Our data reflects real world phenomena over a two-year period, within a large adult palliative home care service with 30 years’ experience locally. The in vivo setting has several implications for our findings. This observational study involving a large dataset posited associations between various factors and after-hours telephone support utilisation. While certain factors were identified to have small but significant correlations, inference of causation was not intended, and their clinical significance remains unsubstantiated. Study findings regarding disposition after a call is received are a reflection of actual practice and not intended as an argument for best practice. It is contingent on the competency and experience of on-call personnel, even though oversight by palliative care specialists is provided throughout.

Next, our codebook is constructed in vivo as there have not been equivalent templates in the literature that fit our purpose; it is used for the first time here and hence this work is the first step towards its validation. With appropriate attention to differences in healthcare setting and culture, it may be applied in similar services like ours where reasons for calling are not systematically documented at point of use, and audits are intentioned.

Lastly, rurality and socio-economic status were not included in this study as factors. As a small and developed city-state, Singapore does not have a rural-urban divide; and residents have almost similar access to healthcare. Besides, home hospice services (including after-hours support) are provided free to all. If the study is replicated elsewhere, these factors may require major consideration.

### Future research

Future work should explore perspectives from service recipients. Specific indicators like support received (or not) and how service had made impact (or otherwise) may be explored through larger sample surveys or smaller qualitative interviews [4, 6]. Service cost implications [13, 14, 16] deserve more attention than received in our study, and we would propose that a formal economic evaluation of the entire after-hours support framework be performed. Results when available will provide valuable guidance to operators and policy makers, as hospice-at-home services are commissioned or funded [17].

## Conclusion

After-hours support remains a vital service element within palliative home care. In our study, almost half of patients supported accessed the service at least once for various reasons; a minority of these calls would be time sensitive while others less so. Characterisation of patient and caller profiles create opportunities for meaningful triage by staff on call. Together with a training package centred around prevalent issues of varying acuity, hospice providers are foreseeably in a better position to render timely and appropriate palliative care 24/7 to patients and caregivers who are supported at home.

## Supplementary Information

**Additional file 1.**

## Data Availability

The datasets generated and/or analysed from this study are available from the corresponding author upon reasonable request.
