# Supplementary material for: Utilisation of after-hours telephone support in a home-based hospice service
Source: BMC Palliat Care. 2022 Sep 16;21:159. doi: 10.1186/s12904-022-01049-5 (PMC9479431; doi:10.1186/s12904-022-01049-5)
Supplement: Supplementary file 1 — Additional file 1. [file 12904_2022_1049_MOESM1_ESM.docx]

# Supplemental file 1: Codebook, sorted in order of Table 4

| Issues | Description & coding guidelines |
| --- | --- |
| Falls or accidents | Of any degree; major or minor |
| Altered Mental State | Restlessness or confusion or agitation only (Drowsiness and lethargy would be coded under Deterioration) |
| Bleeding-related issues | Any type of bleeding (e.g., from tumour, per-rectal or wound bleeding) |
| Fever | Primary issue is the presence of fever, or where the recorded temperate is 37.5 degrees or higher. |
| Medical emergencies | Patient is at risk of sudden and unanticipated death. May include palliative care emergencies and other medical crisis, e.g., hyperglycaemia, complete intestinal obstruction, sudden comatose state. |
| Breathing-related issues | Shortness of breath (SOB); Irregular breathing patterns not related to terminal breathing patterns. |
| Pain | Primary issue is related to pains and aches; include if there are explicit enquiries or advise on the use of morphine or fentanyl (that is not related to shortness of breath) |
| Deterioration or decline | Functional decline not attributed to other causes; may include inability to swallow, noisy secretions, and reduced urine output.  Also, when patient shows signs of entering the terminal phase; may include abnormal breathing patterns and progressive drowsiness or weakness. |
| Sleep-related issues | Include if the primary issue is the patient having trouble sleeping or experiencing sleep reversal. |
| Adverse effects from treatment | Attributed to medications or procedures (e.g., chemotherapy or radiotherapy) |
| Service availability & alternative resource | Primary enquiries are about ambulance service, sale of medication, or asking for private nursing support |
| Diarrhoea | Primary issue includes the explicit mentions of watery stools. |
| Medication Refill | Requests for refill of prescription medication or consumables. |
| Requesting or Donating equipment | Primary reason for the call is to ask for a loan of medical equipment (e.g., oxygen concentrator, hospital bed, commode, wheelchair) or to ask to donate equipment and materials to the organisation. |
| Caller distress | Requiring the professional to address the caller’s emotional or spiritual distress as the primary intervention. |
| Skin-related issues | Primary issue is the presence or management of Oedema, Rashes, Bruises, or other problems related to the skin. |
| Constipation | Primary issue involves explicit enquiries or advice on “constipation”, lack of bowel movement, or use of laxatives. |
| Others | Non-specific issues that do not fit under any other codes; may include symptoms that were not listed, or non-urgent psychosocial issues of any intensity. |
| Administrative matters only | For enquiries regarding visit schedules (e.g., enquiries about visit timings, planning or cancelling home visits) without elaboration.  Also, for updating patient locations with no further elaboration (e.g., informing about hospital admission).  Also, for calls that ask for the patient’s primary nurse without elaboration. |
| Tube-related issues | Primary issue relates to the blockage or displacement of patient’s tubes; also include if issues are related to stomas for tracheostomy tubes or PEG buttons. |
| Informing that patient has died | Informing about patient’s death; to also include requests for home visits to sign death certificate. |
| Clarification or confirmation | For enquiries that is related to clarifying prior instructions or seeking simple guidance to manage patient. |
